# Relating Natural Language Aptitude to Individual Differences in Learning Programming Languages

**DOI:** 10.1038/s41598-020-60661-8

**Published:** 2020-03-02

**Authors:** Chantel S. Prat, Tara M. Madhyastha, Malayka J. Mottarella, Chu-Hsuan Kuo

**Affiliations:** 1 Department of Psychology, University of Washington, Seattle, Washington, USA; 2 Institute for Learning and Brain Sciences, University of Washington, Seattle, Washington, USA; 3 University of Washington Institute for Neuroengineering, Seattle, Washington, USA; 4 Center for Neurotechnology, University of Washington, Seattle, Washington, USA; 5 Department of Radiology, University of Washington, Seattle, Washington, USA

**Keywords:** Language, Working memory, Human behaviour

## Abstract

This experiment employed an individual differences approach to test the hypothesis that learning modern programming languages resembles second “natural” language learning in adulthood. Behavioral and neural (resting-state EEG) indices of language aptitude were used along with numeracy and fluid cognitive measures (e.g., fluid reasoning, working memory, inhibitory control) as predictors. Rate of learning, programming accuracy, and post-test declarative knowledge were used as outcome measures in 36 individuals who participated in ten 45-minute Python training sessions. The resulting models explained 50–72% of the variance in learning outcomes, with language aptitude measures explaining significant variance in each outcome even when the other factors competed for variance. Across outcome variables, fluid reasoning and working-memory capacity explained 34% of the variance, followed by language aptitude (17%), resting-state EEG power in beta and low-gamma bands (10%), and numeracy (2%). These results provide a novel framework for understanding programming aptitude, suggesting that the importance of numeracy may be overestimated in modern programming education environments.

## Introduction

Computer programming has moved from being a niche skill to one that is increasingly central for functioning in modern society. Despite this shift, remarkably little research has investigated the cognitive basis of what it takes to learn programming languages. The implications of such knowledge are wide reaching, both in terms of cultural barriers to pursuing computer sciences^1^ and for educational practices^2^. Central to both are commonly held ideas about what it takes to be a “good” programmer, many of which have not been empirically instantiated.

In fact, remarkably little research has investigated the cognitive bases of “programming aptitude”^3–5^, and, to the best of our knowledge, no research to date has investigated its neural correlates. Critically, the existing research provides inconsistent evidence about the relevance of mathematical skills for learning to program^6–8^. Despite this, programming classes in college environments regularly require advanced mathematical courses as prerequisites. This gap between what we know about learning to program and the environments in which programming is taught was described by Jenkins, who argued that “If computing educators are ever to truly develop a learning environment where all the students learn to program quickly and well, it is vital that an understanding of the difficulties and complexities faced by the students is developed. At the moment, the way in which programming is taught and learned is fundamentally broken”^2^. Unfortunately, little progress has been made since this call to action 15 years ago. Across the same time period, the nature of programming languages has also changed, reducing the likelihood that the original research on learning to program in Pascal^5^ or COBOL^3^, for instance, will generalize to contemporary programming languages.

The research described herein is motivated by a conceptual paradigm shift, namely, that learning to use modern programming languages resembles learning a natural language, such as French or Chinese, in adulthood. Specifically, we argue that research on the neurocognitive bases of programming aptitude has largely missed the fact that computer programming *languages* are designed to resemble the communication structure of the programmer (human languages), an idea that was first formalized by Chomsky over 50 years ago^9^. Although this idea has been revisited in recent reviews^10,11^, only a small number of studies have investigated the predictive utility of linguistic skill for learning programming languages^3,12,13^. Critically, these studies found that natural language ability either predicted unique variance in programming outcomes after mathematical skills were accounted for^3^, or that language was a stronger predictor of programming outcomes than was math^10,11^. Unfortunately these studies are at least thirty years old, and thus reflect both the programming languages and teaching environments of the time.

The current study aimed to fill these gaps by investigating individual differences in the ability to learn a modern programming language through a second language (L2) aptitude lens. Nearly a century of work investigating the predictors of how readily adults learn natural languages has shown that such L2 aptitude is multifaceted, consisting in part of general learning mechanisms like fluid intelligence^14^, working memory capacity^15^, and declarative memory^16,17^, each of which has been proposed to be involved in learning programming languages^4,10^. L2 aptitude has also been linked to more language specific abilities such as syntactic awareness and phonemic coding^16,17^. While the parallels between syntax, or structure building, and learning programming languages are easier to imagine^9,10^, phonemic coding may also be relevant, at least for the vast majority of programming languages which require both producing and reading alphanumeric strings^11^.

We tested the predictive utility of these L2 aptitude constructs for learning to program in Python, the fastest growing programing language in use^18^. The popularity of Python is believed to be driven, in part, by the ease with which it can be learned. Of relevance to our hypothesis, Python’s development philosophy aims to be “reader friendly” and many of the ways in which this is accomplished have linguistic relevance. For instance, Python uses indentation patterns that mimic “paragraph” style hierarchies present in English writing systems instead of curly brackets (used in many languages to delimit functional blocks of code), and uses words (e.g., “not” and “is”) to denote operations commonly indicated with symbols (e.g., “!” and “==”).

To study the neurocognitive bases of learning to program in Python, we recruited 42 healthy young adults (26 females), aged 18–35 with no previous programming experience, to participate in a laboratory learning experiment. In lieu of classroom learning, we employed the Codecademy online learning environment, through which over 4.3 million users worldwide have been exposed to programming in Python. To promote active learning (as opposed to just hitting solution buttons to advance through training), participants were asked to report when and how they asked for help (e.g., hints, forums, or solution buttons), and experimenters verified these reports through screen sharing and screen capture data. Participants were also required to obtain a minimum accuracy of 50% on post-lesson quizzes before advancing to the next lesson. Mean first-pass performance of 80.6% (*SD* = 9.4%) on end-of-lesson quizzes suggests that participants were actively engaged in learning.

Individual differences in the ability to learn Python were assessed using three outcomes (1): learning rate, defined by the slope of a regression line fit to lesson data obtained from each session (2); programming accuracy, based on code produced by learners after training, with the goal of creating a Rock-Paper-Scissors (RPS) game. RPS code was assessed by averaging three raters’ scores based on a rubric developed by a team of expert Python programmers, and the Intraclass Correlation Coefficient (ICC) was calculated as a measure of inter-rater reliability (ICC = 0.996, 95% confidence interval from 0.993–0.998, *F*(35, 70) = 299.41, *p* < 0.001); and (3) declarative knowledge, defined by total accuracy on a 50-item multiple choice test, composed of 25 questions assessing the general purpose of functions, or semantic knowledge (e.g., what does the “str()” method do?) and 25 questions assessing syntactic knowledge (e.g., Which of the following pieces of code is a correctly formatted dictionary?).

The goal of our experiment was to investigate whether factors that predict natural language learning also predict learning to program in Python. To understand the relative predictive utility of such measures, we included factors known to relate to complex skill learning more generally (e.g., fluid reasoning ability, working memory, inhibitory control), and numeracy, the mathematical equivalence of literacy, as predictors. In addition, the current research adopted a neuropsychometric approach, leveraging information about the intrinsic, network-level characteristics of individual brain functioning which have proven to provide unique predictive utility in natural language learning^19–21^. Such an approach allows us to leverage the field of cognitive neuroscience to understand, in a paradigm-free manner, the cognitive bases of learning to program. To investigate these factors, participants completed three behavioral sessions which included standardized assessments of cognitive capabilities as well as a 5-minute, eyes-closed resting-state electroencephalography (rsEEG) recording^20,21^ prior to Python training. The predictive utility of each of these pre-training measures for the three Python learning outcomes were investigated in isolation, and in combination using stepwise regression analyses.

## Results

### Python learning outcomes

**Figure 1:**
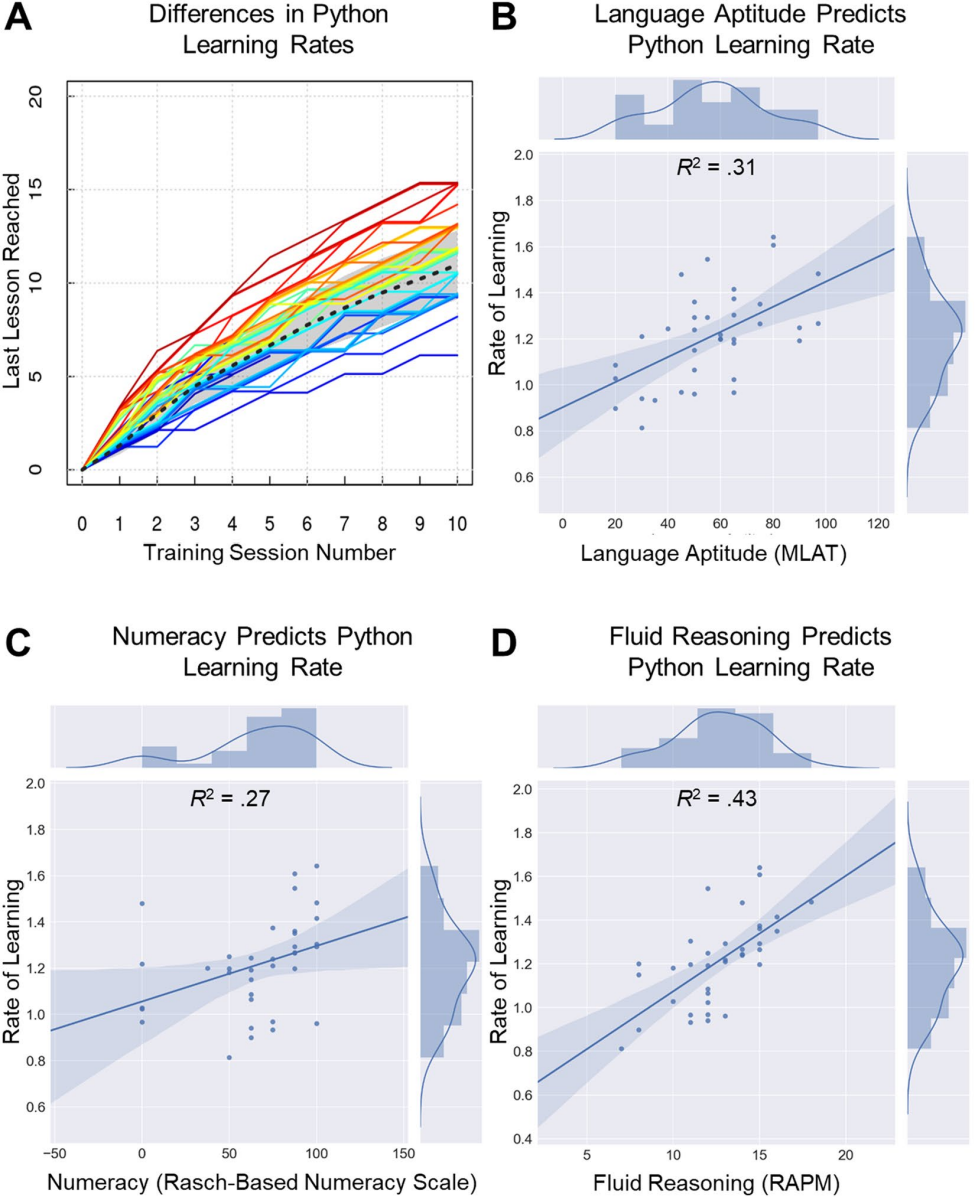
Individual differences in rate of learning to program in Python through Codecademy. (**A**) Individual learning rates computed by regressing last lesson completed during each of 10 training sessions. Each color represents an individual participant, ordered according to the visual light spectrum, ranging from red for the fastest learner, through violet for the slowest. (**B**–**D**) Scatterplots depict the relation between rate of learning on Y axis and (**B**) Language aptitude as measured by the MLAT, (**C**) Numeracy, as measured by the Abbreviated Numeracy Scale, and (**D**) Fluid reasoning, as measured by the Raven’s Advanced Progressive Matrices.

As expected, large individual differences were observed in each of the Python learning outcomes. For example, the fastest learner moved through the lessons two-and-a-half times as quickly as did the slowest learner (Fig. 1A: *mean learning rate* = 1.25, *range* = 0.81–2.0, *sd* = 0.24). Similar variability was also observed in the two post-test measures: programming accuracy (*mean* = 0.57, *range* = 0.01–0.92, *sd* = 0.19) and declarative knowledge test (*mean* = 0.73, *range* = 0.46–0.92, *sd* = 0.10). The three outcome measures were highly positively intercorrelated: learning rate and programming accuracy [*r*(34) = 0.79, *p* < 0.001]; learning rate and declarative knowledge [*r*(34) = 0.74, *p* < 0.001]; and programming accuracy and declarative knowledge [*r*(34) = 0.71, *p* < 0.001]. This provides evidence that people who moved through the program more quickly were not sacrificing speed for learning accuracy.

### Behavioral predictors of Python learning outcomes

#### Language aptitude

Consistent with our hypothesis, language aptitude, as assessed by the Modern Language Aptitude Test (MLAT)^22^, was a robust predictor of all of the Python learning outcomes. Specifically, learning rate [Fig. 1B: *r*(34) = 0.56, *p* < 0.001], programming accuracy [*r*(34) = 0.54, *p* = 0.001], and declarative knowledge [*r*(34) = 0.45, *p* = 0.006] were all positively correlated with MLAT percentile. These correlations remained significant when applying False Discovery Rate (FDR) corrections for multiple comparisons (*p*s <0.05).

#### Numeracy

Numeracy, as measured by the abbreviated numeracy scale^23^, was also a significant predictor of all Python learning outcomes. Specifically, learning rate [Fig. 1C: *r*(29) = 0.52, *p* = 0.003], programming accuracy [*r*(29) = 0.54, *p* = 0.002], and declarative knowledge [*r*(29) = 0.42, *p* = 0.019] were all positively correlated with numeracy scores. Correlations between numeracy and learning rate and programming accuracy remained significant when FDR corrections for multiple comparisons were applied (*p*s < 0.05).

#### General cognitive abilities

Fluid reasoning, working memory updating, working memory span, and inhibitory control, were also significant predictors of learning to program in Python. Specifically learning rate [Fig. 1D: *r*(34) = 0.66, *p* < 0.001], programming accuracy [*r*(34) = 0.71, *p* < 0.001], and declarative knowledge [*r*(34) = 0.55, *p* = 0.001] were all strongly positively correlated with fluid reasoning. Similarly, learning rate [*r*(34) = 0.45, *p* < 0.005], programming accuracy [*r*(34) = 0.54, *p* = 0.001], and declarative knowledge [*r*(34) = 0.41, *p* = 0.013] were all positively correlated with working memory updating. In contrast, working memory span only correlated with learning rate [*r*(34) = 0.43, *p* = 0.01] and programming accuracy [*r*(34) = 0.38, *p* = 0.02], and inhibitory control only correlated with declarative knowledge [*r*(34) = 0.44, *p* = 0.008]. Figure 1 depicts individual differences in rate of learning to program in Python at the group level (A), along with scatterplots relating these differences to language aptitude (B), numeracy (C), and fluid reasoning (D). The complete list of bivariate correlations between behavioral predictors and Python learning outcomes with False Discovery Rate (FDR) corrections applied is included in Supplementary Table S1. Intercorrelations between behavioral predictor variables are included in Supplementary Table S2.

### Resting-state EEG predictors of Python learning outcomes

Our results also provide the first evidence that measures of intrinsic network connectivity obtained from resting-state (rs)EEG can be used to predict Python learning outcomes. The predictors investigated were defined *a priori*, constrained to a set of oscillation-based features of rsEEG that have previously predicted natural language learning^20,21^. The complete set of rsEEG predictors, along with references to the papers in which they relate to natural language learning, are included in Supplementary Tables S3 and S4.

#### rsEEG power

**Figure 2:**
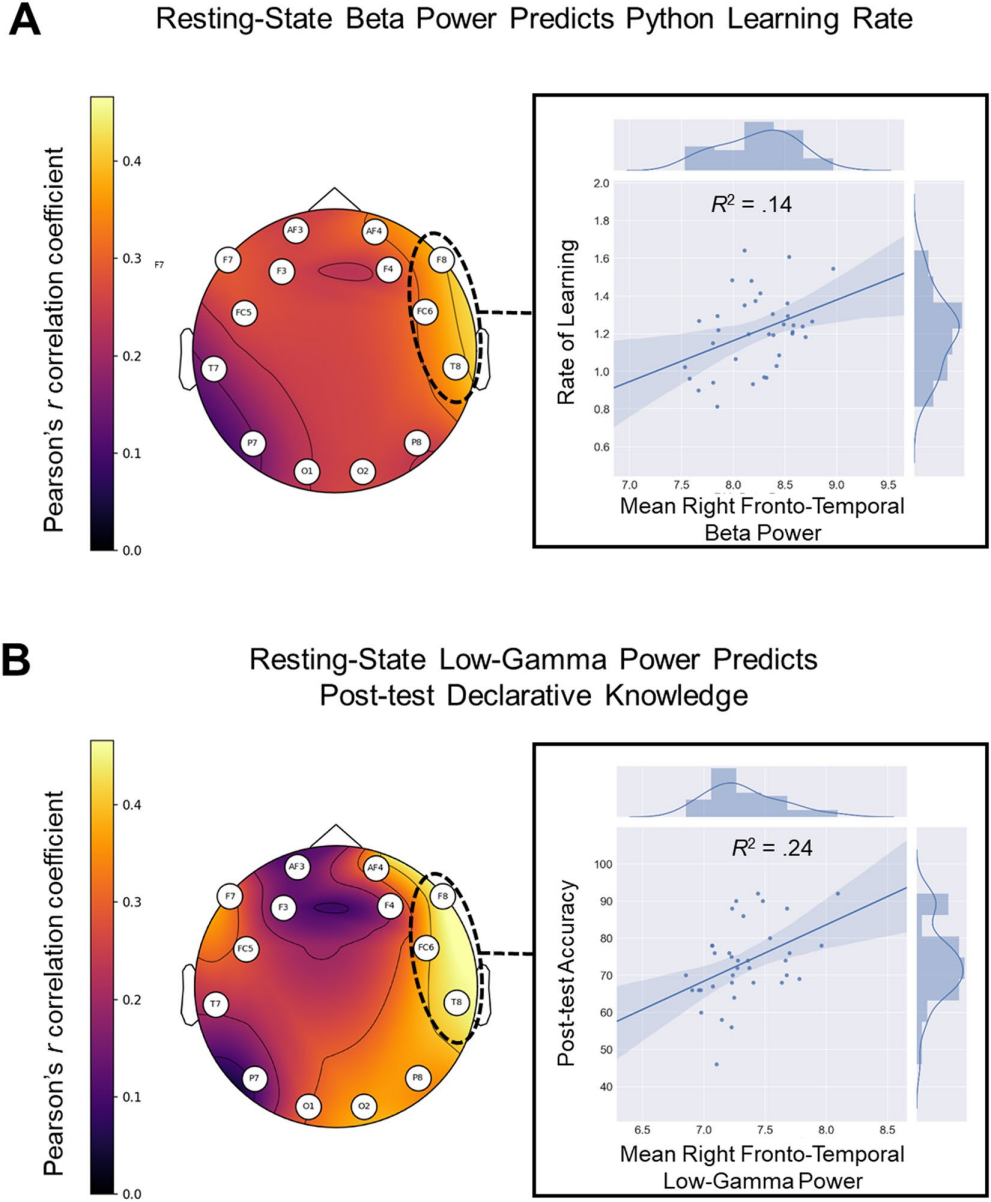
Topomaps displaying the correlations between resting-state EEG power and Python learning outcomes across electrode locations and scatterplots showing data concatenated across right fronto-temporal networks (F8, FC6, T8). (**A**) Correlations between mean beta power (13–29.5 Hz) and Python learning rate across channels, with the relation between mean fronto-temporal beta power and learning rate depicted in scatter plot. (**B**) Correlations between mean low-gamma power (30–40 Hz) and post-test declarative knowledge across channels, with the relation between mean fronto-temporal low-gamma power and declarative knowledge depicted in scatter plot.

Python learning rate was predicted by rsEEG power in the beta frequency range (13–29.5 Hz) recorded over the right fronto-temporal network [Fig. 2A: *r*(35) = 0.39, *p* = 0.020]. Additionally, post-test declarative knowledge was predicted by power in the low-gamma frequency range (30–40 Hz) recorded over the same right fronto-temporal network [Fig. 2B: *r*(35) = 0.43, *p* = 0.009]. The complete set of bivariate correlations between rsEEG power and Python learning outcomes, which did not survive FDR corrections for multiple comparisons, are included in Supplementary Table S3.

#### rsEEG coherence

Only programming accuracy was predicted by rsEEG coherence measures. Specifically, less coherence within the left posterior network was associated with higher programming accuracy across both theta [*r*(34) = −0.37, *p* = 0.031] and beta [*r*(34) = −0.38, *p* = 0.024] frequency bands. The complete set of bivariate correlations between rsEEG power and Python learning outcomes, which did not survive FDR corrections for multiple comparisons, are included in Supplementary Table S4.

### Stepwise regression analyses

To better understand how the cognitive and neural indices investigated combine to predict facile programming in high-aptitude learners, we entered each of the predictors identified by bivariate correlations into separate stepwise regression analyses aimed at explaining the three outcome variables.

#### Learning rate

When the six predictors of Python learning rate (language aptitude, numeracy, fluid reasoning, working memory span, working memory updating, and right fronto-temporal beta power) competed to explain variance, the best fitting model included four predictors: language aptitude, fluid reasoning (RAPM), right fronto-temporal beta power, and numeracy. This model was highly significant [*F*(4,28) = 15.44, *p* < 0.001], and explained 72% of the total variance in Python learning rate. Language aptitude was the strongest predictor, explaining 43.1% of the variance, followed by fluid reasoning, which contributed an additional 12.8% of the variance, right fronto-temporal beta power, which explained 10%, and numeracy scores, which explained 6.1% of the variance.

#### Programming accuracy

By comparison, when the seven predictors of programming accuracy (language aptitude, numeracy, fluid reasoning, working memory span, working memory updating, left posterior theta coherence, left posterior beta coherence) competed for variance, the best fitting model included three predictors: fluid reasoning, language aptitude, and working memory updating. This model was also highly significant [*F*(3,24) = 15.93, *p* < 0.001], and explained 66.7% of the total variance in Python programming accuracy. Fluid intelligence was the strongest predictor of programming accuracy, explaining 50.1% of the total variance, followed by language aptitude, which explained an additional 8.7%, and working memory updating, which explained 7.8% of the variance.

#### Declarative knowledge

Finally, when the eight predictors of post-test declarative knowledge (language aptitude, numeracy, fluid reasoning, working memory updating, inhibitory control, vocabulary, right fronto-temporal low-gamma power, and right posterior low-gamma power) were entered into a stepwise regression analysis, the best fitting model included only two predictors: fluid reasoning and right fronto-temporal-low-gamma power. This model was highly significant [*F*(2,25) = 13.13, *p* < 0.001], and explained 51.2% of the total variance in post-test declarative knowledge scores. Fluid reasoning was also the best predictor of declarative knowledge, explaining 30.9% of the total variance, with right fronto-temporal-low-gamma power explaining an additional 20.3% of the variance.

**Figure 3:**
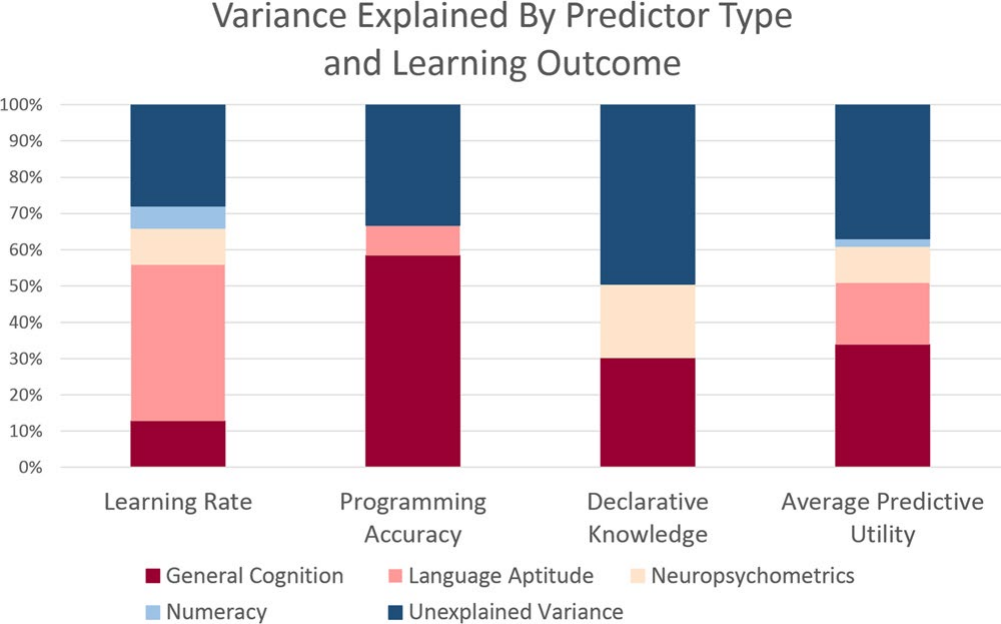
Percentage of total variance explained in stepwise regression analyses of three Python learning outcomes by general cognitive measures (fluid reasoning and working memory), in red, language aptitude (salmon), resting-state EEG (beige), and numeracy (light blue). Unexplained variance is in dark blue. Average predictive utility across outcome variables appears in right-most column.

The results of these regression analyses are summarized visually in Fig. 3, which categorizes the predictor variables into four types: (1) language aptitude; (2) general cognitive; (3) neuropsychometrics; and (4) numeracy.

Full regression tables with fit and uncertainty statistics are included in the supplementary materials. The data and regression scripts, along with readme files that describe them, can be downloaded at https://github.com/UWCCDL/ComputerWhisperers.

## Discussion

The research reported herein describes the first investigation of the neurocognitive predictors of learning to program in Python. The results of this research demonstrate the utility of adopting natural language learning in adulthood as a model for understanding individual differences in the ability to learn modern programming languages. Specifically, using the combination of neural and behavioral measures that have previously been associated with natural language learning, we were able to explain up to 70% of the variability in Python learning outcomes. As depicted in Fig. 3, behavioral language aptitude (salmon) explained an average of over 17% of the variance in Python outcomes (rightmost column). Importantly, either the behavioral indices of language aptitude (salmon), the neural predictors of natural language learning (beige), or both, explained unique variance in Python learning outcomes, even when robust predictors such as fluid reasoning and working memory capacity, which also relate to language learning, were accounted for.

In comparison, numeracy only explained unique variance in Python learning rate, and accounted for an average of 2% of the variance across outcome variables. These results are consistent with previous research reporting higher or unique predictive utility of verbal aptitude tests when compared to mathematical ones^2,12,13^. It is also important to note that some Codecademy lessons focus on computing arithmetic operations, and others use mathematical equations to demonstrate concepts such as “Booleans.” The ability to execute such operations quickly may explain why numeracy predicted unique variance in learning rate, but not in programming accuracy or declarative knowledge.

The regression analyses also showed that general cognitive abilities, including fluid reasoning ability and working memory factors (dark red), were the best average predictors of programming outcomes, explaining nearly 34% of the variance across outcome measures. These results are consistent with the findings of previous research on programming aptitude^4,24^, suggesting that the results generalize to contemporary programming languages as well as to online learning environments. Our results are also consistent with classic information processing models of programming^3^, which include iterative roles for working memory and problem solving. Specifically, these models describe the processes by which programming goals, like language, must be divided into manageable chunks, and the subgoals of these chunks must be held in working memory and used to guide comprehension and production processes^3,10^.

Our findings also provide the first evidence that rsEEG features may be used as neuropsychometric indices of programming aptitude. Specifically, we found that power in beta and low-gamma frequency bands recorded over right fronto-temporal networks at rest predicted unique variance in rate of Python learning and programming accuracy, respectively. In fact, rsEEG measures (Fig. 3: beige) explained an average of 10% of the variance in Python programming outcomes. Similar positive correlations between frontal beta power at rest and learning rate were found in both of our previous rsEEG investigations of natural language learning in adulthood^20,25^. These findings add to the increasing body of literature suggesting that characterizations of resting-state brain networks can be used to understand individual differences in executive functioning^26^ and complex skill learning more broadly^19–21^.

Though a considerable body of research has investigated the relation between individual differences in alpha power at rest and online cognitive processes^27^, the implications of differences in resting-state beta power for cognitive abilities are more mysterious. Beta oscillations, however, have become increasingly associated with *online* language processes^28^. They have also been related to the top-down gating of information into working memory^29^. Similarly, according to the Predictive Coding Framework^30^, beta oscillations function both to *maintain* dynamic representations of meaning during sentence comprehension, and to deploy top-down mechanisms that facilitate comprehension of *predicted* completions. Linking these theories to the current data, one recent study showed that individual differences in the ability to learn syntactic structures in an artificial grammar task were related to differences in synchronization over beta frequencies during learning^31^. A proposed connection between beta computations and resting-state measures is offered by Raichle and Schneider, who suggest that resting-state network activity reflects “… the maintenance of information for interpreting, responding to, and even predicting environmental demands”^32^. Thus, the data reported herein contribute to a growing body of research suggesting that individual differences in beta power and coherence at rest may reflect differences in the ability to acquire and apply statistical knowledge based on sequentially presented information. This proposition is purely speculative and requires further investigations relating differences in resting-state and task-based EEG to learning parameters.

In the current experiment, the various Python learning outcomes were predicted to differing degrees by the components of neurocognitive battery employed. Specifically, programming accuracy was most strongly predicted by fluid cognitive abilities; whereas, learning rate was most strongly predicted by the MLAT, a test that is largely composed of declarative and associative memory-based tasks. These data are consistent with an early cognitive model proposed by Shniederman and Mayer^4^. Specifically, they converge to suggest that learning to program and writing programs depend upon somewhat different cognitive abilities. In particular, both their model and our data highlight the relative importance of analogical reasoning (or problem solving) and working memory capacity for program generation.

Taken together, the results reported herein provide foundational information about the neurocognitive characteristics of “high aptitude” learners of Python, and by virtue, about who may struggle given equal access to learning environments. We argue, as have others before us^2,6^, that both educational and engineering practices have proceeded without this critical knowledge about why, and for whom, learning to program is difficult. Contrary to widely held stereotypes, the “computer whisperers” investigated herein were facile problem solvers with a high aptitude for natural languages. Although numeracy was a reliable predictor of programming aptitude, it was far from the most significant predictor. Importantly, this research also begins the process of identifying the neural characteristics of individual differences in Python learning aptitude, which can be used as targets for technologies such as neurofeedback and neurostimulation that modify patterns of connectivity and alter corresponding behaviors^33,34^. Important future work is needed to determine the extent to which our results will translate to classroom learning environments, to less “user friendly” languages such as Java that are more widely employed in software engineering spaces, and to higher programming proficiency levels. Still, the research reported herein begins to paint a picture of what a good programmer *actually* looks like, and that picture is different in important ways from many previously held beliefs.

## Methods

### Participants

Forty-two healthy adults aged 18–35 years were recruited for participation in this study. Five participants were excluded from these analyses due to attrition (not completing the training sessions), and one participant was excluded because he was an extreme outlier in learning rate (>3 *sd* away from the mean). The remaining 36 participants (21 female) were included in our analyses. All participants were right-handed native English speakers with no exposure to a second natural language before the age of 6 years (*mean* = 13.9 years, *range* = 6–22 years), and with moderate self-rated second language proficiency (*mean* = 3.2/10, range = 0–8/10). All experimental protocols and paradigms were approved by the University of Washington Institutional Review Board, and all participants provided informed consent in accordance with the standards set forth by the same review board. All individuals were compensated for their participation.

### Materials

#### Rasch-based numeracy scale

Numeracy was assessed using a Rasch-Based Numeracy Scale which was created by evaluating 18 numeracy questions across multiple measures and determining the 8 most predictive items^23^. The test was computerized and untimed.

#### Raven’s advanced progressive matrices (RAPM)

This study used a shortened 18-item version of this task, which was developed by splitting the original 36 questions into two, difficulty-matched, subtests based on the data reported in^35^. These parallel forms were previously used in our natural language aptitude research^20,25^.

#### Simon task

The Simon task is a non-verbal measure designed to assess susceptibility to stimulus-response interference. The version used herein, which consisted of 75% congruent and 25% incongruent trials, has been previously used to model individual differences in complex skill learning^36,37^.

#### 3-Back task

The N-Back Task is a measure designed to index working memory updating. In the 3-back version of the task, participants are presented with a stream of letters and are tasked with determining if the presented letter is the same or different from the letter presented three items ago. Participants respond “Same” or “Different” by pressing one of two designated buttons. Total task accuracy was calculated out of 80 items, and used as a metric of working memory updating.

#### The probabilistic stimulus selection task (PSS)

The PSS task is an implicit learning task which measures individual differences in sensitivity to positive and negative feedback^38^. Sensitivity to positive feedback (Choose Accuracy) and negative feedback (Avoid Accuracy) are calculated independently and used as measures of interest.

#### Attentional blink task (AB)

The AB task is a measure of the temporal limitations of attention. In this task, participants are shown a rapid stream of serially presented letters, and are told to attend to two numbers embedded within the letter stream. The lag between the offset of the first number and the onset of the second number is varied such that the second number falls either inside (100–500 ms) or outside (<100 ms or >500 ms) the attentional blink window. The AB task used herein has been previously related to differences in language experience^39^.

#### Complex working memory span tasks

Computerized Reading Span, Operation Span, and Symmetry Span^40,41^ were used to index working memory capacity. A single composite score was computed by z-transforming each individual span score and taking an average of the three scores.

#### Modern language aptitude test (MLAT)

All participants completed the MLAT^16^, a standardized measure of second-language aptitude normed for native English speaking adults. The MLAT is a well-validated measure which has been shown to predict up to 30% of variability in language learning^42^.

### Procedures

#### Pre-test

Participants completed a comprehensive psychometric battery, which was divided into three 1.5 hour behavioral testing sessions and a rsEEG recording. The order of these pre-test sessions was counterbalanced across participants.

#### Python training sessions

Participants completed ten 45-minute learning sessions using Codecademy’s Learn Python course. The course is composed of interactive modules designed for users that have no prior coding experience. Each module consists of lessons that focus on key concepts of coding in Python (e.g., lists, dictionaries, if statements). These concepts are explicitly tested at the end of most modules through multiple choice quizzes. To advance to the next exercise, participants were required to pass the quizzes with an accuracy of 50% or greater. When faced with challenges during learning, participants were able to use the help resources available to them, but all such behaviors were reported to, and recorded by the experimenter, who shared a mirror screen with the participant. When “stuck”, participants were instructed to use the help resources in the following order: (1) Hint Button: a generic hint pertaining to a given exercise, (2) Community Forum: a help blog where participants are able to view questions and responses from other users of Codecademy, and (3) Solution Button: a button which corrects the participant’s code. At the end of each training session, the participant’s “place” in the lesson was recorded. This data was used both to calculate learning rate and to ensure that they started in that same place when beginning their next session.

#### Post-test

Following Python training, participants completed a one-hour posttest session consisting of a multiple-choice declarative knowledge test and a coding production test.

### rsEEG analysis

Five-minutes of eyes-closed rsEEG were collected at 128 Hz using a wireless 14-channel headset (Emotiv EPOC). Data analysis procedures are outlined in^20^ and the code for these analyses is publicly available in the following github repository (https://github.com/UWCCDL/QEEG).

### Python outcome measures

#### Learning rate

Learning rate was calculated by fitting a regression line to terminal-level data at each session with the intercept fixed at zero. The slope of this line is operationalized as a given participant’s learning rate.

#### Declarative knowledge

Following the ten session Python training, participants completed a 50-item multiple-choice test consisting of information presented in the first 14 lessons in Codecademy. Half of the questions queried semantic knowledge, or an understanding of the purpose of functions in Python, and the other half investigated syntactic knowledge including questions such as “Which line of code is formatted correctly?” Participants were given 30 minutes to complete this test.

#### Programming accuracy

After completing the multiple choice portion of the post-test, participants were tasked with programming a Rock-Paper-Scissors (RPS) game that a user could play against the computer. This project was created by Codecademy, but participants did not complete it during learning. The programming test included instructions that partially broke down the larger problem into predefined steps. Participants were not allowed to use any additional resources (e.g., notes, forums, help buttons) to complete the project but were able to run and test their code. Participants were given 30 minutes to complete the project. The code they produced was scored by three independent raters using a rubric developed by Python experts that assigned points to each step of the project (ICC = 0.996, 95% confidence interval from 0.993–0.998, *F*(35,70) = 299.41, *p* < *0*. 001). Programming accuracy was operationalized as each participant’s score out of 51 total points.

## Supplementary information

Supplementary Materials .

## Data Availability

All data is available in the manuscript, supplementary materials, or on github. Data not on repositories will be made available upon request.

## References

[1] Cheryan S, Ziegler SA, Montoya AK, Jiang L (2017). Why are some STEM fields more gender balanced than others?. Psychol. Bull..

[2] Jenkins T (2002). On the difficulty of learning to program. Proceedings of the 3rd Annual Conference of the LTSN Centre for Information and Computer Sciences.

[3] Sauter VL (1986). Predicting computer programming skill. Comput. Educ..

[4] Shneiderman B, Mayer R (1979). Syntactic/semantic interactions in programmer behavior: a model and experimental results. Int. J. Comput. Inf. Sci..

[5] Shute VJ (1991). Who is likely to acquire programming skills?. J. Educ. Comput. Res..

[6] Davy, J. & Jenkins, T. Research-led innovation in teaching and learning programming. *Proceedings of the 4th Annual Conference on Innovation and Technology in Computer Science Education*, 5–8 (1999).

[7] Stein MV (2002). Mathematical preparation as a basis for success in CS-II. J. Comput. Sci. Coll..

[8] Quille, K. & Bergin, S. Programming: predicting student success early in CS1. a re-validation and replication study. *Proceedings of the 23*^*rd*^*Annual ACM Conference on Innovation and Technology in Computer Science Educati**on 2018*, 15–20 (2018).

[9] Chomsky N (1956). Three models for the description of language. IRE T. Inform. Theor..

[10] Federenko, E., Ivanova, A., Dhamala, R. & Bers, M.U. The Language of Programming: A Cognitive Perspective. *Trends in Cognitive Sciences* (2019). 10.1016/j.tics.2019.04.010 31153775

[11] Vee A (2013). Understanding computer programming as a literacy. *Literacy in Composition*. Studies.

[12] Austin HS (1987). Predictors of pascal programming achievement for community college students. ACM SIGCSE Bulletin.

[13] Leeper RR, Silver JL (1982). Predicting success in a first programming course. ACM SIGCSE Bulletin.

[14] Gardner RC, Lambert WE (1965). Language aptitude, intelligence, and second-language achievement. J. of Educ. Psychol..

[15] Miyake, A. & Friedman, N. P. Individual differences in second language proficiency: Working memory as language aptitude. *Foreign language learning: Psycholinguistic studies on training and retention*, 339–364 (1998).

[16] Carroll, J. & Sapon, S. Modern Language Aptitude Test. San Antonio, TX: Psychological Corporation (1959).

[17] Dörnyei, Z. The psychology of second language acquisition. Oxford, UK: Oxford University Press (2009).

[18] Heath, N. How one programming language is leaving rivals in the dust. *Tech Republic*, https://www.techrepublic.com/article/how-one-programming-language-is-leaving-rivals-in-the-dust/ (2019).

[19] Chai XJ (2016). Intrinsic functional connectivity in the adult brain and success in second-language learning. J. Neurosci..

[20] Prat CS, Yamasaki BL, Kluender RA, Stocco A (2016). Resting-state qEEG predicts rate of second language learning in adults. Brain Lang..

[21] Prat, C. S. & Yamasaki, B. L. Resting-state qEEG reveals intrinsic network differences between monolingual and bilingual adults. The Bilingual Brain, A Lifelong Perspective, Quebec City, Canada (2018).

[22] Carroll, J. B. & Sapon, S. M. *Modern Language Aptitude Test* (Psychological Corporation, 1959).

[23] Weller JA (2013). Development and testing of an abbreviated numeracy scale: a Rasch analysis approach. J. Behav. Decis. Making.

[24] Kurtz BL (1980). Investigating the relationship between the development of abstract reasoning and performance in an introductory programming class. ACM SIGCSE Bulletin.

[25] Prat CS, Yamasaki BL, Peterson ER (2019). Individual differences in resting-state brain rhythms uniquely predict second language learning rate and willingness to communicate in adults. J. Cognitive Neurosci..

[26] Reineberg AE, Andrews-Hanna JR, Depue BE, Friedman NP, Banich MT (2015). Resting-state networks predict individual differences in common and specific aspects of executive function. Neuroimage.

[27] Klimesch W (1999). EEG alpha and theta oscillations reflect cognitive and memory performance: a review and analysis. Brain Research Reviews.

[28] Weiss S, Mueller HM (2012). Too many betas do not spoil the broth: the role of beta brain oscillations in language processing. Frontiers in Psychol..

[29] Miller EK, Lundqvist M, Bastos AM (2018). Working memory 2.0. Neuron.

[30] Lewis AG, Schoffelen JM, Schriefers H, Bastiaansen M (2016). A predictive coding perspective on beta oscillations during sentence-level language comprehension. Frontiers in Human Neurosci..

[31] Kepinska O, Pereda E, Caspers J, Schiller NO (2017). Neural oscillatory mechanisms during novel grammar learning underlying language analytical abilities. Brain and Lang..

[32] Raichle ME, Snyder AZ (2007). A default mode of brain function: A brief history of an evolving idea. Neuroimage.

[33] Zoefel B, Huster RJ, Herrmann CS (2011). Neurofeedback training of the upper alpha frequency band in EEG improves cognitive performance. Neuroimage.

[34] Enriquez-Geppert S, Huster RJ, Herrmann CS (2013). Boosting brain functions: improving executive functions with behavioral training, neurostimulation, and neurofeedback. Int. J. Psychophysiol..

[35] Arthur W, Day D (1994). Development of a short form for the Raven’s Advanced Progressive Matrices Test. Educ. Psychol. Meas..

[36] Stocco A (2017). Individual differences in the Simon effect are underpinned by differences in competitive dynamics in the basal ganglia: An experimental verification and a computational model. Cognition.

[37] Stocco A, Yamasaki BL, Prat CS (2018). Human performance across decision making, selective attention, and working memory tasks: Experimental data and computer simulations. Data Brief..

[38] Frank MJ, Seeberger LC, O’reilly RC (2004). By carrot or by stick: cognitive reinforcement learning in parkinsonism. Science.

[39] Yamasaki BL, Stocco A, Prat CS (2018). Relating individual differences in bilingual language experience to executive attention. Lang. Cog. Neurosci..

[40] Oswald FL, McAbee ST, Redick TS, Hambrick DZ (2015). The development of a short domain-general measure of working memory capacity. Behav. Res. Methods.

[41] Foster JL (2015). Shortened complex span tasks can reliably measure working memory capacity. Mem. Cognition.

[42] Ehrman, M. A study of the Modern Language Aptitude Test for predicting learning success and advising students. *Language Aptitude Invitational Symposium Program Proceedings*, 74–99 (1994).

