# Supplementary material for: Relating Natural Language Aptitude to Individual Differences in Learning Programming Languages
Source: Sci Rep. 2020 Mar 2;10:3817. doi: 10.1038/s41598-020-60661-8 (PMC7051953; doi:10.1038/s41598-020-60661-8)
Supplement: Supplementary file 1 — Supplementary Materials . [file 41598_2020_60661_MOESM1_ESM.docx]

Supplementary Materials for

Relating Natural Language Aptitude to Individual Differences in Learning Programming Languages

Chantel S. Prat^1,2,3*^, Tara M. Madhyastha^4^, Malayka J. Mottarella^1,2^, Chu-Hsuan Kuo^1^

Correspondence to: [csprat@uw.edu](mailto:csprat@uw.edu)

**Bivariate Correlations between Python Learning Outcomes and Behavioral Measures**

|  | Learning Rate | Programming  Accuracy | Declarative Knowledge |
| --- | --- | --- | --- |
| Language Aptitude (MLAT) | 0.560** | 0.541** | 0.449** |
| Numeracy | 0.515** | 0.542** | 0.418** |
| Fluid Reasoning (RAPM) | 0.657** | 0.714** | 0.547** |
| Working Memory Span (Composite Score) | 0.425** | 0.382** | 0.135 |
| Working Memory Updating (3-Back Acc) | 0.454** | 0.538** | 0.409** |
| Inhibition (Simon RT) | 0.238 | 0.175 | 0.441** |
| Implicit Learning (PSS Choose Acc) | 0.017 | -0.052 | 0.158 |
| Implicit Learning (PSS Avoid Acc) | 0.070 | 0.041 | 0.135 |
| Attentional Blink Size | 0.112 | 0.013 | 0.253 |

**Supplementary Table S1.**

Bivariate correlations of Python outcome variables and all behavioral predictor variables. Pearson’s *r* values with * = *p* < 0.05 uncorrected, ** = *p* < 0.05 FDR corrected.

**Inter-correlations among Python Learning Outcomes and Behavioral Measures**

|  | Language Aptitude (MLAT) | Numeracy | Fluid  Reasoning (RAPM) | WM Span  (Composite  Span Score) | WM Updating  (3-Back) |
| --- | --- | --- | --- | --- | --- |
| Language Aptitude (MLAT) |  |  |  |  |  |
| Numeracy | 0.285 |  |  |  |  |
| Fluid Reasoning | 0.485** | 0.600** |  |  |  |
| WM Span | 0.394** | 0.348 | 0.299 |  |  |
| WM Updating | 0.376** | 0.624** | 0.542** | 0.398** |  |
| Inhibition | 0.069 | 0.286 | 0.135 | -0.263 | 0.148 |

**Supplementary Table S2.**

Bivariate correlations between all significant behavioral predictor variables. Pearson’s *r* values with * = *p* < 0.05 uncorrected, ** = *p* < 0.05 FDR corrected.

**Bivariate Correlations between Python Learning Outcomes and EEG Power Indices**

|  | Predictor Source | Learning Rate | Programming Accuracy | Declarative Knowledge |
| --- | --- | --- | --- | --- |
| Left Frontotemporal Beta | 1, 2 | 0.269 | 0.268 | 0.297 |
| Right Frontotemporal Beta | 1, 2 | 0.385* | 0.315 | 0.304 |
| Left Posterior Beta | 1 | 0.206 | 0.078 | 0.113 |
| Right Posterior Beta | 1, 2 | 0.275 | 0.315 | 0.223 |
| Left Frontotemporal Gamma | 1 | 0.213 | 0.150 | 0.298 |
| Right Frontotemporal Gamma | 1, 2 | 0.308 | 0.161 | 0.429* |
| Left Posterior Gamma | 1 | 0.208 | 0.035 | 0.213 |
| Right Posterior Gamma | 1, 2 | 0.144 | 0.208 | 0.326 |
| Medial Frontal Gamma | 1 | 0.258 | 0.115 | 0.211 |

Supplementary Table S3.

Bivariate correlations between rsEEG network power predictors and Python outcome variables with predictor sources: (1) Prat, Yamasaki, Kluender, & Stocco, 2016; (2) Prat, Yamasaki, & Peterson, (2018). Pearson’s *r* values with * = *p* < 0.05 uncorrected, ** = *p* < 0.05 FDR corrected.

**Bivariate Correlations between Python Learning Outcomes and EEG Coherence**

|  | Learning Rate | Programming Accuracy | Declarative Knowledge |
| --- | --- | --- | --- |
| Right Frontotemporal Alpha Coherence | 0.236 | 0.055 | 0.136 |
| Left Posterior  Alpha Coherence | -0.242 | -0.334* | -0.273 |
| Right Frontotemporal  Beta Coherence | 0.015 | 0.028 | -0.224 |
| Left Frontotemporal  Beta Coherence | -0.210 | -0.380* | -0.227 |
| Right Frontotemporal Theta Coherence | -0.037 | -0.042 | 0.027 |
| Left Posterior  Theta Coherence | -0.258 | -0.366* | -0.297 |
| Right Frontotemporal Gamma Coherence | -0.024 | -0.083 | -0.164 |
| Left Posterior  Gamma Coherence | -0.083 | -0.230 | -0.154 |
| Right Frontotemporal to Posterior Alpha Coherence | -0.024 | 0.067 | 0.019 |
| Right Frontotemporal to Posterior Theta Coherence | 0.037 | 0.172 | 0.017 |

**Supplementary Table S4.**

Bivariate correlations between rsEEG network-level coherence predictors and Python outcome variables defined based on Prat, Yamasaki, & Peterson, (2018). Pearson’s *r* values with * = *p* < 0.05 uncorrected, ** = *p* < 0.05 FDR corrected.

**Predicting Learning Rate: Model Summary**

| Model | Variable Added | R^2^ | ΔR^2^ | F (df) | *p* | BIC | ΔBIC | Evid. |
| --- | --- | --- | --- | --- | --- | --- | --- | --- |
| 1 | Language Aptitude | 0.431 |  | 20.47 (1, 27) | <0.001 | -15.592 |  |  |
| 2 | Fluid Intelligence | 0.559 | 0.128 | 16.46 (2, 26) | <0.001 | -19.586 | -3.994 | + |
| 3 | Right Fronto-Temporal Beta Power | 0.659 | 0.100 | 16.08 (3, 25) | <0.001 | -23.665 | -4.079 | + |
| 4 | Numeracy | 0.720 | 0.061 | 15.44 (4, 24) | <0.001 | -26.061 | -2.396 | + |
|  |  |  |  |  |  |  |  |  |
| 5a | Working Memory Span | 0.720 | 0.000 | 11.84 (5, 23) | <0.001 | -22.695 | 3.366 | - |
| 5b | Working Memory Updating | 0.722 | 0.002 | 11.96 (5, 23) | <0.001 | -22.916 | 3.145 | - |

**Supplementary Table S5.**

Summary of stepwise regression analyses predicting Learning Rate, with incremental addition of variables at each step. R^2^ uncorrected, F with degrees of freedom in parenthesis, p value, and Bayesian Information Criterion (BIC) for each model are included on each row. ΔR^2^ and ΔBIC shows the change in these variables from the previous step. Evid. = Evidence for a better fitting model based ΔBIC from Raftery (1995); +positive, ++strong, +++very strong, and – for no evidence. Dashed line indicates the point at which adding any additional variable did not increase the fit of the model. Subsequent models (5a and 5b) show statistics comparing models that include each remaining variable to the best fitting model (4).

**Predicting Learning Rate: Parameter Estimates**

|  | Model 1 | | | | | |
| --- | --- | --- | --- | --- | --- | --- |
| Predictors | β | b | SE (b) | 95% CI (b) | t | *p* |
| Intercept |  | 0.848 | 0.086 | [0.669, 1.028] | 9.693 | <0.001 |
| Language Aptitude | 0.657 | 0.006 | 0.001 | [0.003, 0.009] | 4.524 | <0.001 |
|  | Model 2 | | | | | |
| Intercept |  | 0.565 | 0.130 | [0.298, 0.832] | 4.350 | <0.001 |
| Language Aptitude | 0.442 | 0.004 | 0.001 | [0.001, 0.007] | 2.909 | 0.007 |
| Fluid Intelligence | 0.417 | 0.032 | 0.117 | [0.008, 0.056] | 2.741 | 0.011 |
|  | Model 3 | | | | | |
| Intercept |  | -0.919 | 0.561 | [-2.073, 0.236] | -1.639 | 0.114 |
| Language Aptitude | 0.412 | 0.004 | 0.001 | [0.001, 0.007] | 3.012 | 0.006 |
| Fluid Intelligence | 0.394 | 0.030 | 0.010 | [0.009, 0.052] | 2.889 | 0.008 |
| Right Fronto-Temporal Beta Power | 0.319 | 0.185 | 0.068 | [0.044, 0.325] | 2.705 | 0.012 |
|  | Model 4 | | | | | |
| Intercept |  | -1.289 | 0.543 | [-2.409, -0.169] | -2.376 | 0.026 |
| Language Aptitude | 0.419 | 0.004 | 0.001 | [0.002, 0.007] | 3.314 | 0.003 |
| Fluid Intelligence | 0.174 | 0.013 | 0.012 | [-0.012, 0.038] | 1.096 | 0.284 |
| Right Fronto-Temporal Beta Power | 0.381 | 0.220 | 0.065 | [0.086, 0.354] | 3.391 | 0.002 |
| Numeracy | 0.331 | 0.382 | 0.166 | [0.039, 0.726] | 2.297 | 0.031 |

**Supplementary Table S6.**

Parameter estimates of models predicting Learning Rate at all significant steps of the stepwise regression. β = standardized beta, b = unstandardized beta, SE = standard error.

**Predicting Programming Accuracy: Model Summary**

| Model | Variable Added | R^2^ | ΔR^2^ | F (df) | *p* | BIC | ΔBIC | Evid. |
| --- | --- | --- | --- | --- | --- | --- | --- | --- |
| 1 | Fluid Intelligence | 0.501 |  | 26.13 (1, 26) | <0.001 | -19.981 |  |  |
| 2 | Language Aptitude | 0.588 | 0.087 | 17.85 (2, 25) | <0.001 | -22.008 | -2.027 | + |
| 3 | Working Memory Updating | 0.666 | 0.078 | 15.93 (3, 24) | <0.001 | -24.523 | -2.515 | + |
|  |  |  |  |  |  |  |  |  |
| 4a | Numeracy | 0.669 | 0.003 | 11.60 (4, 23) | <0.001 | -21.427 | 3.096 | - |
| 4b | Working Memory Span | 0.682 | 0.016 | 12.35 (4, 23) | <0.001 | -22.621 | 1.902 | - |
| 4c | Left Posterior Beta Coherence | 0.687 | 0.021 | 12.62 (4, 23) | <0.001 | -23.036 | 1.487 | - |
| 4d | Left Posterior Theta Coherence | 0.680 | 0.014 | 12.24 (4, 23) | <0.001 | -22.445 | 2.078 | - |

**Supplementary Table S7.**

Summary of stepwise regression analyses predicting Programming Accuracy outcome, with incremental addition of variables at each step. R^2^ uncorrected, F with degrees of freedom in parenthesis, p value, and Bayesian Information Criterion (BIC) for each model are included on each row. ΔR^2^ and ΔBIC shows the change in these variables from the previous step. Evid. = Evidence for a better fitting model based ΔBIC from Raftery (1995); +positive, ++strong, +++very strong, and – for no evidence. Dashed line indicates the point at which adding any additional variable did not increase the fit of the model. Subsequent models (4a – 4d) show statistics comparing models that include each remaining variable to the best fitting model (3).

**Predicting Programming Accuracy: Parameter Estimates**

|  | Model 1 | | | | | |
| --- | --- | --- | --- | --- | --- | --- |
| Predictors | β | b | SE (b) | 95% CI (b) | t | *p* |
| Intercept |  | -0.087 | 0.133 | [-0.360, 0.186] | -0.657 | 0.517 |
| Fluid Intelligence | 0.708 | 0.053 | 0.010 | [0.032, 0.074] | 5.111 | <0.001 |
|  | Model 2 | | | | | |
| Intercept |  | -0.112 | 0.123 | [-0.366, 0.143] | -0.904 | 0.375 |
| Fluid Intelligence | 0.536 | 0.040 | 0.011 | [0.017, 0.063] | 3.612 | 0.001 |
| Language Aptitude | 0.341 | 0.003 | 0.001 | [0.0003, 0.006] | 2.297 | 0.030 |
|  | Model 3 | | | | | |
| Intercept |  | -0.583 | 0.230 | [-1.057, -0.109] | -2.538 | 0.018 |
| Fluid Intelligence | 0.311 | 0.023 | 0.012 | [-0.003, 0.049] | 1.862 | 0.075 |
| Language Aptitude | 0.361 | 0.003 | 0.001 | [0.0007, 0.006] | 2.637 | 0.015 |
| Working Memory Updating | 0.353 | 0.796 | 0.337 | [0.100, 1.491] | 2.361 | 0.027 |

**Supplementary Table S8.**

Parameter estimates of models predicting Learning Rate at all significant steps of the stepwise regression. β = standardized beta, b = unstandardized beta, SE = standard error.

**Predicting Declarative Knowledge: Model Summary**

| Model | Variable Added | R^2^ | ΔR^2^ | F (df) | *p* | BIC | ΔBIC | Evid. |
| --- | --- | --- | --- | --- | --- | --- | --- | --- |
| 1 | Fluid Intelligence | 0.309 |  | 11.63 (1, 26) | 0.002 | -47.932 |  |  |
| 2 | Right Fronto-Temporal Gamma Power | 0.512 | 0.203 | 13.13 (2, 25) | <0.001 | -54.352 | -6.42 | ++ |
|  |  |  |  |  |  |  |  |  |
| 3a | Language Aptitude | 0.547 | 0.035 | 9.654 (3, 24) | <0.001 | -53.078 | 1.274 | - |
| 3b | Numeracy | 0.531 | 0.019 | 9.065 (3, 24) | <0.001 | -52.128 | 2.224 | - |
| 3c | Working Memory Updating | 0.522 | 0.010 | 8.475 (3, 24) | <0.001 | -51.601 | 2.751 | - |
| 3d | Inhibitory Control | 0.565 | 0.053 | 10.39 (3, 24) | <0.001 | -54.229 | 0.123 | - |
| 3e | Vocabulary | 0.554 | 0.042 | 9.93 (3, 24) | <0.001 | -53.511 | 0.841 | - |
| 3f | Right Posterior Low Gamma Power | 0.515 | 0.003 | 8.49 (3, 24) | <0.001 | -51.165 | 3.187 | - |

**Supplementary Table S9.**

Summary of stepwise regression analyses predicting Declarative Knowledge outcome, with incremental addition of variables at each step. R^2^ uncorrected, F with degrees of freedom in parenthesis, p value, and Bayesian Information Criterion (BIC) for each model are included on each row. ΔR^2^ and ΔBIC shows the change in these variables from the previous step. Evid. = Evidence for a better fitting model based ΔBIC from Raftery (1995); +positive, ++strong, +++very strong, and – for no evidence. Dashed line indicates the point at which adding any additional variable did not increase the fit of the model. Subsequent models (3a – 3f) show statistics comparing models that include each remaining variable to the best fitting model (2).

**Predicting Declarative Knowledge: Parameter Estimates**

|  | Model 1 | | | | | |
| --- | --- | --- | --- | --- | --- | --- |
| Predictors | β | b | SE (b) | 95% CI (b) | t | *p* |
| Intercept |  | 0.475 | 0.080 | [0.310, 0.639] | 5.927 | <0.001 |
| Fluid Intelligence | 0.556 | 0.021 | 0.006 | [0.008, 0.034] | 3.410 | 0.002 |
|  | Model 2 | | | | | |
| Intercept |  | -0.645 | 0.354 | [-1.373, 0.083] | -1.824 | 0.080 |
| Fluid Intelligence | 0.507 | 0.019 | 0.005 | [0.008, 0.030] | 3.611 | 0.001 |
| Right Fronto-Temporal Gamma Power | 0.453 | 0.156 | 0.048 | [0.056, 0.254] | 3.228 | 0.003 |

**Supplementary Table S10.**

Parameter estimates of models predicting Learning Rate at all significant steps of the stepwise regression. β = standardized beta, b = unstandardized beta, SE = standard error.
